# Kinetics of intestinal ultrasound and shear-wave elastography to assess early response in ulcerative colitis patients treated with filgotinib

**DOI:** 10.1093/ecco-jcc/jjaf185

**Published:** 2025-10-28

**Authors:** Maarten Jan Pruijt, Christoph Teichert, Floris Antonius De Voogd, Reimer Jacques Janssen, Mark Löwenberg, Rogier Leon Goetgebuer, Geert Renaat D’Haens, Krisztina Barbara Gecse

**Affiliations:** Department of Gastroenterology and Hepatology, Amsterdam UMC, University of Amsterdam, Amsterdam, The Netherlands; Department of Gastroenterology and Hepatology, Amsterdam UMC, University of Amsterdam, Amsterdam, The Netherlands; Department of Gastroenterology and Hepatology, Amsterdam UMC, University of Amsterdam, Amsterdam, The Netherlands; Department of Gastroenterology and Hepatology, Amsterdam UMC, University of Amsterdam, Amsterdam, The Netherlands; Department of Gastroenterology and Hepatology, Amsterdam UMC, University of Amsterdam, Amsterdam, The Netherlands; Department of Gastroenterology and Hepatology, Amsterdam UMC, University of Amsterdam, Amsterdam, The Netherlands; Department of Gastroenterology and Hepatology, Amsterdam UMC, University of Amsterdam, Amsterdam, The Netherlands; Department of Gastroenterology and Hepatology, Amsterdam UMC, University of Amsterdam, Amsterdam, The Netherlands

**Keywords:** intestinal ultrasound (IUS), ulcerative colitis (UC), filgotinib, shear-wave elastography (SWE)

## Abstract

**Background and Aims:**

A reduction of bowel wall thickness (BWT) on intestinal ultrasound (IUS) predicts endoscopic response in ulcerative colitis (UC). Advanced techniques such as shear-wave elastography (SWE) might enhance response assessment. We aimed to identify early IUS parameters to predict treatment response to filgotinib in UC.

**Methods:**

This prospective observational study included UC patients with endoscopically active disease (endoscopic Mayo score [EMS] ≥2, extending beyond the rectum) starting on filgotinib. IUS parameters, including SWE, were assessed at baseline (T0), week 4 (T1), and at second endoscopy (T2). EMS of both the most-affected segment and the sigmoid was assessed at T0 and T2, and endoscopic response was defined a ≥1 point decrease in EMS.

**Results:**

A total of 23 patients were included. Six of 21 patients who underwent a second endoscopy were endoscopic responders. At T1 in the sigmoid, a BWT decrease of ≥1.33 mm or ≥24.7% (odds ratio [OR]: 32.5 [2.4-443.2], *P *= .009 and OR: 13.8 [1.2-156.6], *P *= .035) and submucosa decrease of ≥20.8% (OR: 13.8 [1.2-156.6], *P *= .035) predicted endoscopic response. Additionally, color Doppler signal (CDS) improvement at T1 predicted endoscopic response (OR: 20.0 [1.7-241.7], *P *= .018). In the sigmoid, SWE values changed differently over time between responders and non-responders (T2: 9.9 ± 15.7 vs −8.1 ± 11.4 kPa, *P *= .002). However, SWE values at T1 were not predictive of endoscopic response (OR: 1.07 [0.99-1.16], *P *= .088).

**Conclusions:**

On IUS, BWT, submucosal thickness, and CDS predict endoscopic response after 4 weeks of filgotinib treatment. SWE values in the sigmoid differ between responders and non-responders, but early assessment does not predict treatment response.

## Graphical Abstract

**Graphical Abstract:**
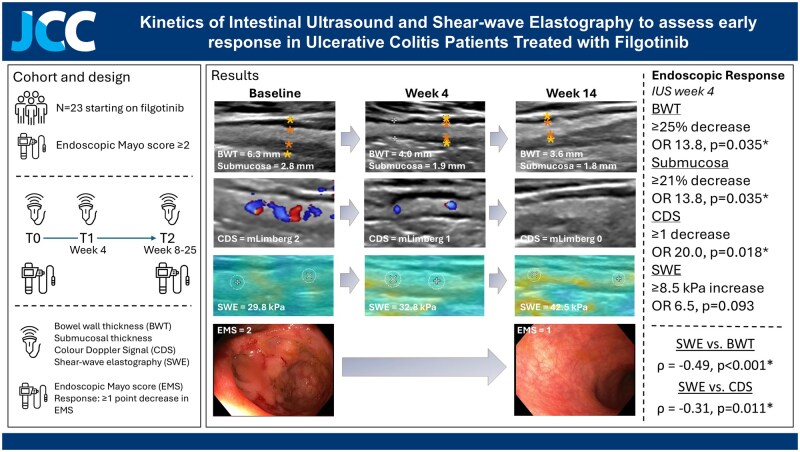

## 1. Introduction

Ulcerative colitis (UC) is a chronic inflammatory disease that affects the colon, and is characterized by a relapsing–remitting pattern.^1^ The clinical management of UC often involves a trial-and-error approach as tools are lacking to predict treatment response.^2^ Mucosal healing, as assessed by endoscopy, is considered one of the treatment goals in UC. However, endoscopy is invasive, expensive, and time-consuming, which limits its utility as a frequent tool for monitoring disease activity.^3–6^ Additionally, growing evidence indicates that UC involves transmural changes, highlighting the need for assessment beyond the mucosa alone.^7^^,^^8^ Intestinal ultrasound (IUS) is a fast, cost-effective, and non-invasive imaging tool for assessing UC activity, which can be used in a point-of-care setting by measuring bowel wall thickness (BWT) as the most important parameter.^9–12^

A recent study demonstrated that BWT at 6 weeks after initiating anti-inflammatory agents predicts endoscopic response and remission.^13^ Previous studies have also demonstrated different sonographic dynamics for various therapeutic agents, with the pan-JAK inhibitor tofacitinib showing a significant BWT decrease as early as week 2.^12–14^ However, sample sizes for the individual groups were too small to draw firm conclusions.

Filgotinib, a preferential JAK1 inhibitor, is effective in inducing and maintaining clinical remission in UC.^15^ IUS changes during filgotinib treatment have not been characterized previously.

In addition to conventional B-mode IUS, advanced modalities such as shear-wave elastography (SWE) may aid the assessment of bowel wall composition by analyzing tissue stiffness. SWE provides a quantitative measure of bowel stiffness, potentially distinguishing inflammation from fibrosis.^16^ While several studies have explored the role of elastography in CD, data on its use in UC remain scarce.^17–19^ In this study, we aimed to identify if early IUS, including SWE, could serve as a valid surrogate marker for treatment response to filgotinib in moderate to severe UC.

## 2. Methods

### 2.1. Study design

In this prospective, observational, single-center study, consecutive adult patients with an established diagnosis of UC were eligible for inclusion. Patients with moderate to severe UC (ie, endoscopic Mayo score [EMS] ≥2, extending beyond the rectum) starting on filgotinib treatment, as per discretion of the treating physician, were included. Patients with proctitis only, ongoing gastroenteritis, history of colectomy or imminent need for colectomy, and pregnancy were excluded. All patients provided informed consent. This study was approved by the medical ethics committee of the Amsterdam University Medical Center. Data were collected using a logged data management system (Castor EDC).

### 2.2. Procedures

All patients received filgotinib 200 mg once daily. As per standard of care, patients were evaluated before starting treatment (T0), after 4 weeks (T1) of treatment, and at the time of the second endoscopy between week 8 and 25 (T2). Age, gender, height, smoking status, and medical history were collected at T0. At all visits, weight, modified Mayo score (MMS),^20^ serum C-reactive protein (CRP), and fecal calprotectin (FCP) levels were assessed.

#### 2.2.1. Intestinal ultrasound

IUS examinations of all colonic segments were performed by a single sonographer (M.P., >3 years of experience) at T0, T1, and T2. IUS was not performed on the same day of the endoscopy. Filgotinib was initiated either on the same day as the T0 IUS or within 2 weeks without any treatment changes. The window between IUS and endoscopy could not exceed 4 weeks and treatment was not changed between IUS and endoscopy. IUS examinations were performed using an Epiq 5G ultrasound scanner (Philips) with C5-1, L12-5 and L18-4 probes. Gain and focus settings were optimized per patient for optimal image quality. A velocity scale of 5 cm/s was used to measure color Doppler signal (CDS). All colonic segments were visualized starting at the sigmoid and moving proximally to the ascending colon. Two separate cine-loops for B-mode parameters and two for CDS were recorded for every colonic segment in a longitudinal and cross-sectional plane. SWE involved 10 consecutive measurements in the submucosal layer of the sigmoid colon in a longitudinal plane.^21^ For the assessment of submucosal echogenicity, a longitudinal still image of the sigmoid was taken.

#### 2.2.2. Endoscopy

Patients underwent a complete endoscopy or flexible sigmoidoscopy at T0 and at the time of response evaluation (T2), as per local guidelines or guided by the discretion of the treating physician. During endoscopy, withdrawal of the procedure was recorded.

### 2.3. Parameters

#### 2.3.1. Intestinal ultrasound

All B-mode IUS parameters are given in Table S1. For SWE, the lowest and highest measurements were excluded to correct for outliers, and the average of the eight remaining measurements was calculated.^21^ Submucosal echogenicity was quantified using the relative submucosal echogenicity (RSE), calculated from the mean aerial grayscale intensity (a numerical scale from 0 to 255, with zero corresponding to black) in a longitudinal still image of the sigmoid.^8^ To account for variations in depth and gain, we measured the difference in grayscale intensity between the submucosa and muscularis propria in a perpendicular fashion. Each measurement was performed twice within one still image with ≥10 mm between measurements, and RSE was calculated as ((2× measurement submucosa)/2)−((2× measurement muscularis propria)/2).

In cases of wall layer stratification loss, adjacent segments with preserved layers were used to identify the muscularis propria and submucosa for accurate RSE and SWE assessments.

All B-mode IUS parameters, including RSE, were scored for each segment using a DICOM-viewer (RadiAnt DICOM Viewer). At all time points, the UC-IUS index was calculated based on the B-mode parameters: BWT, CDS, loss of haustrations, and presence of fatty wrapping.^22^

All pseudonymized cine loops were stored and measured >4 weeks after the last visit of the last patient by one sonographer (M.P.). A second reader (C.T., >2 years of experience) who was blinded for clinical, biochemical, and endoscopic data, read 30 randomly selected IUS examinations and scored all B-mode IUS parameters for the sigmoid colon. The results from the first reader (M.P.) were used for further analysis. The results from the second reader (C.T.) were used to assess inter-observer agreement.

#### 2.3.2. Endoscopy

Once recruitment was completed, two blinded expert gastroenterologists (G.D. and M.L.) independently reviewed all endoscopic videos in random order. Each reader assessed the videos, scoring the most severe segment using the EMS. Additionally, the rectum and sigmoid were scored separately using the EMS. In cases of disagreement, a third reader (K.G.) adjudicated by selecting one of the initial scores. If the disagreement exceeded 1 point on the EMS, the third reader determined the final score, which could differ from the scores provided by the initial two readers.

#### 2.3.3. Definitions and outcomes

The primary objective of the study was to investigate whether changes in any IUS measurements (B-mode, CDS, and SWE) between T0 and T1 could predict endoscopic endpoints, as defined by the EMS at T2. The primary outcome was the difference in BWT at T1 predicting endoscopic endpoints at T2. The secondary outcomes included differences for all IUS parameters (B-mode and SWE) and the UC-IUS index between clinical, biochemical, and endoscopic endpoints at T1 and T2. Additionally, all IUS parameters were correlated to endoscopic severity.

Clinical remission was defined as MMS ≤2 with all subscores ≤1. Clinical response was defined as MMS decrease of ≥3.^6^ Biochemical remission was defined as having both CRP ≤5 mg/L and FCP ≤150 mg/kg. Endoscopic remission was defined as EMS=0, endoscopic improvement as EMS ≤1, and endoscopic response as a decrease of EMS ≥1^6^ (Table S2).

#### 2.3.4. Sample size calculation

We performed a sample size calculation to detect a significant difference in BWT at T1 to differentiate between patients with endoscopic improvement versus no endoscopic improvement. In tofacitinib-treated patients, a recent study found a difference in BWT of the sigmoid colon of 2.35 ± 1.12 mm, between responders (patients with endoscopic improvement, EMS ≤1, at T2) and non-responders.^13^ With a level of significance of α = .05 and 80% power, five patients in the responder group were needed to detect this difference. When assuming 17%-34% of patients show endoscopic improvement,^15^ 20 patients will be needed. Using a 10% drop-out rate, 22 patients had to be included.

#### 2.3.5. Statistical analysis

SPSS Statistics for Windows, v.28 (IBM Corp.) was used for statistical analysis. Mean ± SD and median with interquartile range (IQR) were used to report normally and non-normally distributed data. A (paired) *t* test, Mann–Whitney *U* test, Wilcoxon rank test, chi-square test, or McNemar test were used as appropriate. Area under the receiver-operating characteristic curve (AUROC) was used to determine accuracy, sensitivity, specificity, positive predictive value (PPV), and negative predictive value (NPV). Optimal cut-offs were determined from the AUROC using the Youden index, and additional high-specificity thresholds were explored to minimize false positives. Odds ratios (ORs) were calculated with logistic regression analysis. Spearman’s correlation coefficient was used to determine negligible (0.00-0.09), weak (0.10-0.39), moderate (0.40-0.69), strong (0.70-0.89), or very strong (0.90-1.00) correlation.^23^ Intraclass correlation coefficient and Cohen’s kappa (weighted when ≥3 categories) statistics were used to determine interobserver agreement. A *P-*value of .05 was used to determine significance.

## 3. Results

In total, 23 patients were included (Table 1). Ten patients were female (10/23, 44%) and the mean disease duration was 14.4 years (SD ± 10.4). Fourteen patients (14/23, 61%) failed two or more biologics, 16 patients (16/23, 70%) were previously exposed to anti-tumor necrosis factor (anti-TNF) treatment, and one patient (1/23, 4%) received tofacitinib prior to filgotinib treatment. Eight patients (8/23, 35%) were receiving concomitant systemic corticosteroids at T0. Of these, six patients were still on corticosteroids at T1, and four continued corticosteroid treatment through T2.

**Table 1.** Baseline characteristics.

Baseline characteristics	All patients (*n* = 23)	Responders (*n* = 6)	Non-responders (*n* = 15)
**Female**	10 (44%)	2 (33%)	7 (47%)
**Age at inclusion in years (mean±SD)**	42.7 ± 14.0	43.3 ± 12.4	44.0 ± 14.6
**Age at diagnosis in years (mean±SD)**	28.4 ± 9.7	28.5 ± 12.1	29.9 ± 8.5
**Disease duration in years (mean±SD)**	14.4 ± 10.4	14.8 ± 13.1	14.1 ± 9.5
**BMI in kg/m^2^ (mean±SD)**	23.3 ± 3.5	25.2 ± 3.3	23.2 ± 3.3
**Montreal classification**			
** Left-sided colitis (E2)**	10 (43%)	2 (33%)	8 (53%)
** Pancolitis (E3)**	13 (57%)	4 (67%)	7 (47%)
**Biological or JAK-i exposure**			
** None**	4 (17%)	1 (17%)	2 (13%)
** One**	5 (22%)	1 (17%)	4 (27%)
** Two**	8 (35%)	2 (33%)	6 (40%)
** Three**	6 (26%)	2 (33%)	3 (20%)
**Anti-TNF exposure**	16 (70%)	4 (67%)	11 (73%)
**JAK-i exposure**	1 (4%)	1 (17%)	—
**Concomitant treatment(s)**			
** Systemic 5-ASA**	12 (52%)	6 (100%)	5 (33%)
** Rectal 5-ASA**	6 (26%)	2 (33%)	3 (20%)
** Systemic corticosteroids**	8 (35%)	1 (17%)	6 (40%)
** Rectal corticosteroids**	1 (17%)	1 (17%)	2 (13%)
**Clinical and biochemical parameters**			
** Modified Mayo score (mean±SD)**	6.2 ± 1.9	6.0 ± 1.4	6.3 ± 2.2
** CRP in mg/L (median, IQR)**	3.7 (1.9-9.0)	3.4 (1.3-22.2)	4.6 (2.3-9.0)
** Fecal calprotectin in mg/kg (median, IQR)**	662 (134-3135)	1898 (270-6000)	726 (158-2725)
**Intestinal ultrasound parameters sigmoid**			
** Bowel wall thickness in mm (mean±SD)**	4.38 ± 1.29	4.27 ± 0.74	4.57 ± 1.47
** Mucosa thickness in mm (mean±SD)**	1.20 ± 0.37	1.31 ± 0.39	1.20 ± 0.38
** Submucosa thickness in mm (mean±SD)**	1.91 ± 0.86	1.68 ± 0.45	2.02 ± 1.00
** Muscularis propria in mm (mean±SD)**	1.27 ± 0.41	1.29 ± 0.27	1.35 ± 0.42
** CDS (mLimberg ≥1)**	22 (96%)	6 (100%)	14 (93%)
** Loss of haustrations**	15 (65%)	5 (83%)	9 (60%)
** Loss of stratifications**	4 (17%)	1 (17%)	2 (13%)
** Presence of fatty wrapping**	18 (78%)	5 (83%)	11 (73%)
** Presence of lymph nodes**	6 (26%)	2 (33%)	3 (20%)
** Shear-wave elastography in kPa (median, IQR)**	30.01 (24.9-42.5)	32.51 (28.3-42.5)	29.88 (23.2-37.8)
**Endoscopic Mayo score**			
** eMayo 0**	—	—	—
** eMayo 1**	1 (4%)	—	1 (7%)
** eMayo 2**	9 (39%)	—	7 (47%)
** eMayo 3**	13 (57%)	6 (100%)	7 (47%)
**Days between IUS and first dose (median, IQR)**	0 (0-1)	0 (0-10)	0 (0-3)
**Days between IUS and endoscopy (median, IQR)**	8 (2-29)	7 (4-15)	22 (2-35)
**Weeks between T0 and T2 (median, IQR)**	14 (12-17)	14 (12-16)	13 (11-20)

Abbreviations: UC, ulcerative colitis; SD, standard deviation; BMI, body mass index; JAK-i, JAK inhibitor; CRP, C-reactive protein; IQR, interquartile range; CDS, color Doppler signal; T0, baseline, T2, weeks 8-25.

All outcomes are depicted in Table S2. At T2, nine patients were clinical responders (9/21, 43%) and three patients were in clinical remission (3/21, 14%). Biochemical remission was observed in three patients (3/19, 16%).

A total of 21 patients (21/23, 91%) underwent a follow-up endoscopy between 8 and 25 weeks (mean 14.6 ± 4.8 weeks). Two patients declined a follow-up endoscopy. Median time between IUS and endoscopy at T2 was 2 days (IQR 0-6.5). In all patients at both time points, the worst segment on endoscopy was either the sigmoid or the rectum. At T2, six patients (6/21, 29%) were classified as endoscopic responders, four (4/21, 19%) achieved endoscopic improvement, and no patient (0/21, 0%) reached complete endoscopic remission.

### 3.1. Baseline IUS parameters

At T0, all B-mode parameters showed no significant differences between endoscopic responders and non-responders (Tables 2 and 3).

**Table 2.** Continuous IUS variables in the sigmoid between responders (EMS decrease of ≥1) and non-responders at all time points.

Continuous variables in the sigmoid	Endoscopic response (*n* = 6)	Endoscopic non-response (*n* = 15)	*P*-value
**Baseline (T0)**			
**BWT (mm)**	4.57±1.5	4.27±0.7	.648
**Submucosa (mm)**	1.68±0.4	2.02±1.0	.445
**SWE (kPa)**	34.1±7.3	32.7±11.5	.787
**RSE (grayscale value)**	74.9 ± 39.4	94.4 ± 42.1	.341
**T1**			
**BWT (mm)**	2.75±1.2	3.99±1.7	.125
**BWT decrease (mm)**	1.52±1.3	0.57±0.9	.064
**BWT decrease (%)**	35.1±31.0	14.7±22.4	.106
**Submucosa (mm)**	1.02±0.5	1.81±1.0	.022
**Submucosa decrease (mm)**	0.67±0.4	0.21±0.4	.042
**Submucosa decrease (%)**	40.0±23.9	11.5±24.5	.025
**SWE (kPa)**	40.5±12.7	32.3±9.0	.115
**SWE change (kPa)**	6.3±12.6	-0.4±15.6	.366
**SWE change (%)**	20.1 (IQR −17.0 to 48.2)	1.11 (IQR −29.7 to 26.8)	.340
**RSE (grayscale value)**	86.2 ± 32.5	101.5 ± 46.4	.475
**T2**			
**BWT (mm)**	2.09 (IQR 1.8-2.5)	4.10 (IQR 2.2-5.6)	.023*
**BWT decrease (mm)**	2.10±0.6	0.47±1.4	.006*
**BWT decrease (%)**	48.8±10.2	10.7±25.9	.001*
**Submucosa (mm)**	0.73 (IQR 0.7-1.0)	1.93 (IQR 0.8-2.6)	.055
**Submucosa decrease (mm)**	0.88±0.3	0.23±0.5	.007*
**Submucosa decrease (%)**	51.3±10.3	12.8±23.6	.001*
**SWE (kPa)**	38.6±11.0	31.5±10.7	.189
**SWE change (kPa)**	4.5±14.6	−1.1±14.6	.430
**SWE change (%)**	4.7 (IQR −22.1 to 77.3)	10.12 (IQR −27.6 to 38.0)	.470
**RSE (grayscale value)**	66.3 ± 56.9	98.0 ± 34.1	.130

Abbreviations: EMS, endoscopic Mayo score; BWT, bowel wall thickness; SWE, shear-wave elastography; RSE, relative submucosal echogenicity; T0, baseline; T1, week 4; T2, follow-up endoscopy; IQR, interquartile range.*P < .05

**Table 3.** Categorical IUS variables in the sigmoid between responders (endoscopic Mayo score decrease of ≥1) and non-responders at all time points.

Categorical variables in the sigmoid	Endoscopic response (*n* = 6)	Endoscopic non-response (*n* = 15)	*P*-value
**Baseline (T0)**			
**CDS mLimberg 0**	0 (0%)	1 (7%)	.173
**Loss of haustration**	5 (83%)	9 (60%)	.613
**Loss of stratification**	1 (17%)	2 (13%)	1.000
**Fatty wrapping**	5 (83%)	11 (73%)	1.000
**Presence of lymph nodes**	2 (33%)	3 (20%)	.598
**T1**			
**CDS mLimberg 0**	4 (67%)	1 (7%)	.011*
**CDS decrease ≥1**	5 (83%)	3 (20%)	.014*
**UC-IUS < 5**	5 (83%)	5 (33%)	.063
**UC-IUS decrease ≥3**	5 (83%)	1 (7%)	.002*
**Loss of haustration**	0 (0%)	7 (47%)	.072
**Normalization haustration**	4 (67%)	1 (7%)	.002*
**Loss of stratification**	0 (0%)	1 (7%)	1.000
**Fatty wrapping**	2 (33%)	9 (60%)	.351
**Presence of lymph nodes**	0 (0%)	2 (13%)	1.000
**T2**			
**CDS mLimberg 0**	6 (100%)	4 (27%)	.004*
**CDS decrease ≥1**	6 (100%)	6 (40%)	.019*
**UC-IUS <4**	5 (83%)	6 (40%)	.149
**UC-IUS decrease ≥4**	5 (83%)	2 (13%)	.006*
**Loss of haustration**	1 (17%)	8 (53%)	.178
**Normalization haustration**	4 (67%)	2 (13%)	.031*
**Loss of stratification**	1 (17%)	1 (7%)	.500
**Fatty wrapping**	1 (17%)	9 (60%)	.149
**Presence of lymph nodes**	0 (0%)	3 (20%)	.526

Abbreviations: T0, baseline; T1, week 4; T2, follow-up endoscopy; CDS, color Doppler signal; UC-IUS, ulcerative colitis intestinal ultrasound index.*P < .05

### 3.2. Changes in IUS parameters at T1

#### 3.2.1. BWT and submucosal thickness at T1 in endoscopic responders

**Figure 1.**
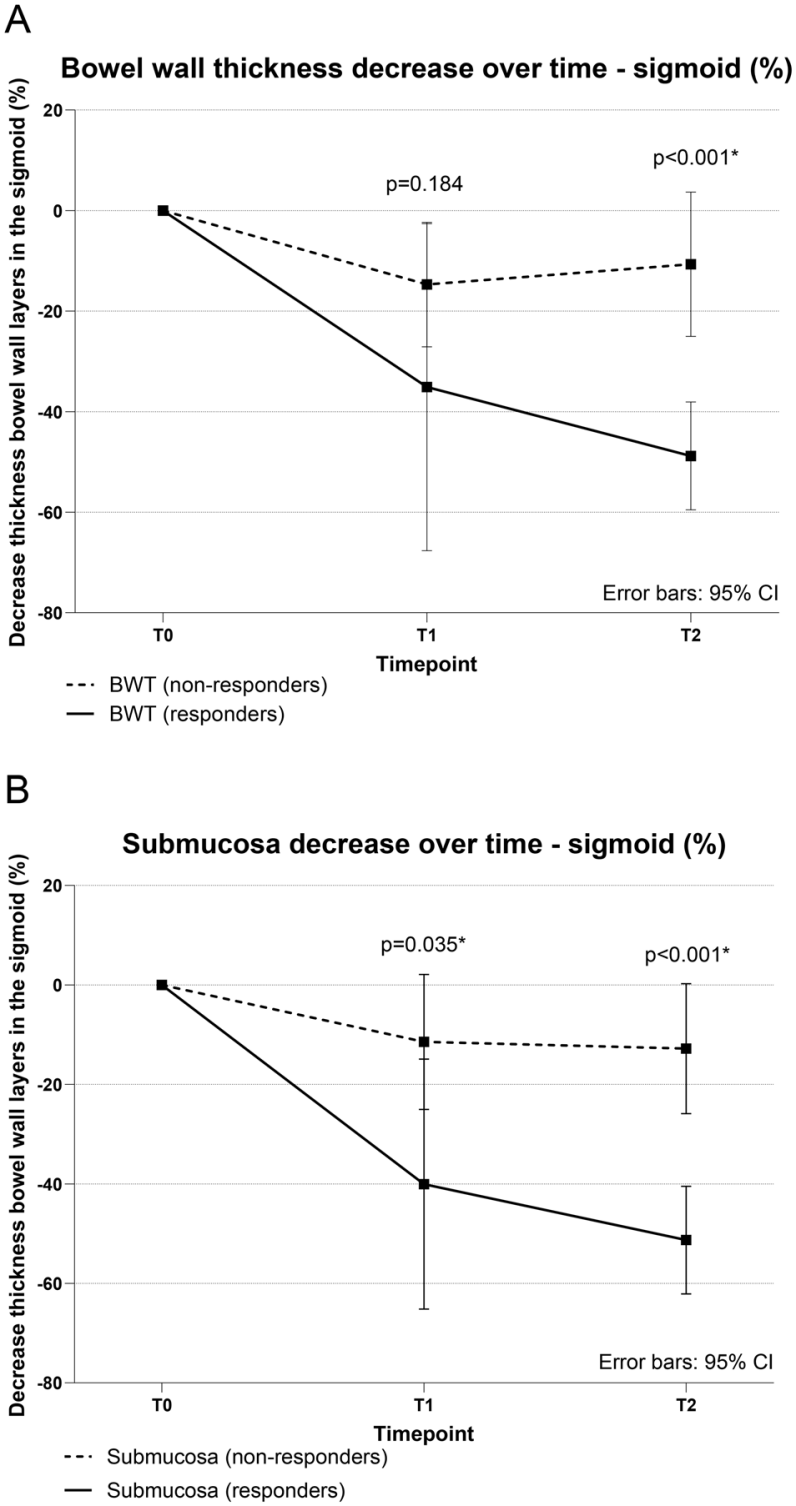
Percentage change in bowel wall thickness (A) and submucosa (B) over time in responders and non-responders (endoscopic Mayo score decrease of ≥1). BWT, bowel wall thickness; CI, confidence interval; T0, baseline; T1, week 4; T2, follow-up endoscopy.

In the sigmoid colon at T1, the mean BWT (2.75 ± 1.2 mm vs 3.99 ± 1.7 mm, *P *= .125; Table 2) and the millimeter and percentage decrease in BWT compared to T0 (1.52 ± 1.3 mm vs 0.57 ± 0.9 mm, *P *= .064 and 35.1 ± 31% vs 14.7 ± 22% *P *= .106, respectively; Figure 1A, Table 2) were not significantly different between endoscopic responders and non-responders.

**Figure 2.**
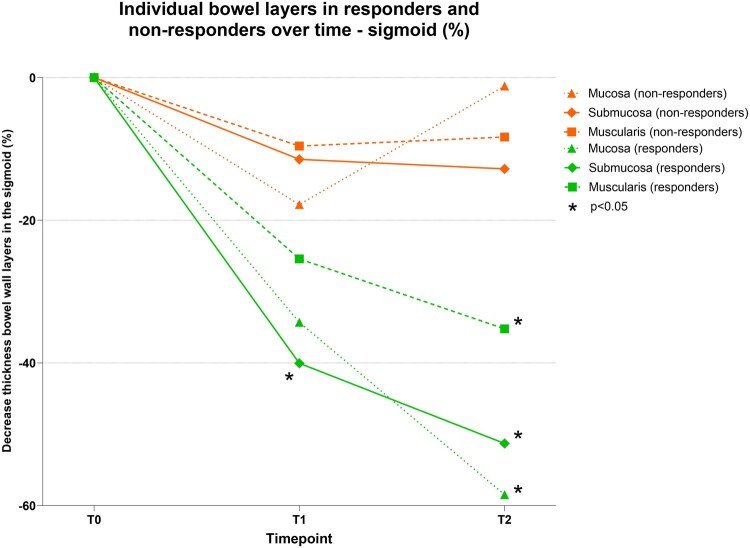
Decrease in thickness of all individual bowel wall layers in the sigmoid over time between endoscopic responders (EMS decrease of ≥1) and non-responders. *A significant difference (*P *< .05) for the specific bowel wall layer thickness between responders and non-responders at that time point. EMS, endoscopic Mayo score; T0, baseline; T1, week 4; T2, follow-up endoscopy.

Mean submucosal thickness was significantly lower in endoscopic responders compared to non-responders at T1 (1.02 ± 0.5 mm vs 1.81 ± 1.0 mm, *P *= .022). Compared to T0, the millimeter and percentage decrease in submucosal thickness were significantly higher from T1 onwards in endoscopic responders (0.67 ± 0.4 mm vs 0.21 ± 0.4 mm, *P *= .042 and 40 ± 24% vs 11.5 ± 24.5%, *P *= .025; Figure 1B, Table 2). Other individual bowel wall layers, including the mucosa and the muscularis propria, were not significantly different between endoscopic responders and non-responders at T1 (Figure 2).

#### 3.2.2. Other B-mode parameters of the sigmoid at T1 in endoscopic responders

At T1, a higher percentage of patients with endoscopic response demonstrated a decrease of at least one point in CDS (83% vs 20%, *P *= .014; Table 3). Colonic haustration was normalized in all responders at this time point (loss of haustration 0% vs 47%, *P *= .072; Table 3). Additionally, there were no patients with any loss of stratification and presence of lymph nodes in the responder group (0% vs 7%, *P *= 1.00 and 0% vs 13%, *P *= 1.00, respectively; Table 3).

#### 3.2.3. Optimal cut-offs at T1 to predict endoscopic response

**Figure 3.**
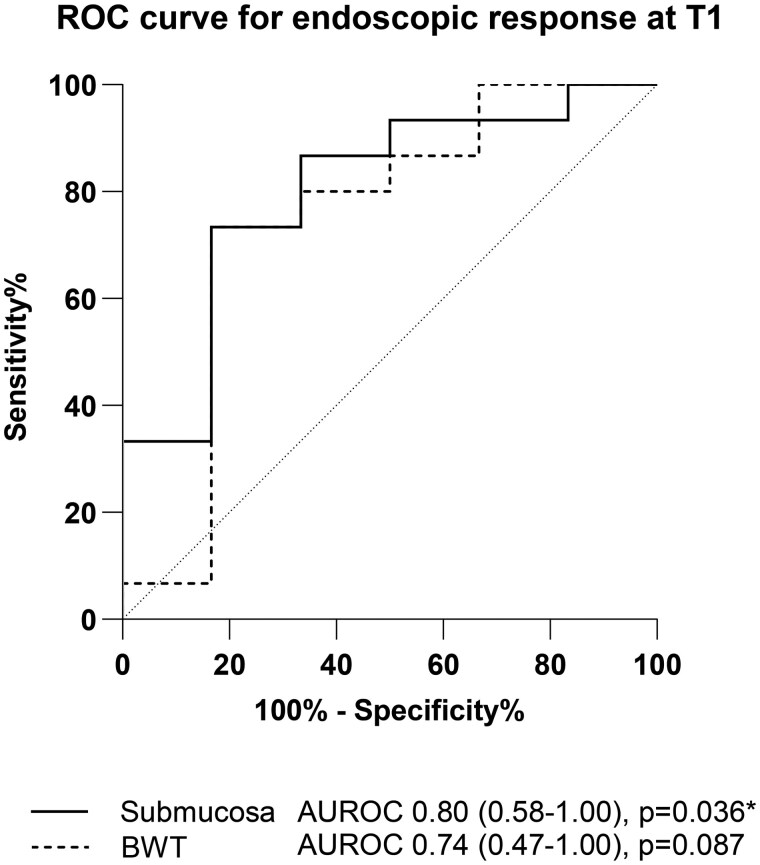
ROC curve analysis for endoscopic response (endoscopic Mayo score decrease of ≥1) of bowel wall thickness and submucosal thickness decrease in percentage at T1. ROC, receiver operating characteristics curve; BWT, bowel wall thickness; T0, baseline; T1, week 4; T2, follow-up endoscopy; AUROC, area under the receiver operating characteristic curve.

A decrease of ≥1.33 mm BWT at T1 compared to T0 demonstrated endoscopic response with 83% sensitivity, 87% specificity, 71% PPV, and 93% NPV (AUROC: 0.79, 95% CI 0.51-1.00, *P *= .043; Table 4). A percentage decrease of ≥24.72% BWT at T1 demonstrated endoscopic response with 83% sensitivity, 73% specificity, 56% PPV, and 92% NPV (AUROC: 0.74, 95% CI 0.47-1.00, *P *= .087; Figure 3, Table 4). For the submucosal layer, a percentage reduction of ≥20.76% demonstrated endoscopic response with 83% sensitivity, 73% specificity, 56% PPV and 92% NPV (AUROC: 0.80, 95% CI 0.58-1.00, *P *= .036; Figure 3, Table 4). Additionally, a decrease of ≥3 points in the UC-IUS index at T1 compared to T0 demonstrated endoscopic response with 83% sensitivity, 93% specificity, 83% PPV, and 93% NPV (AUROC: 0.82, 95% CI 0.54-1.00, *P *= .024; Table 4).

**Table 4.** ROC curve analyses for endoscopic response (endoscopic Mayo score decrease of ≥1).

	AUC	*P*-value	Cut-off(s)	Sens	Spec
**Sigmoid T1**					
**BWT T1 (mm)**	0.700 (0.47-0.93)	.161	3.22 mm	0.667	0.667
**BWT decrease T1 (mm)**	0.789 (0.51-1.00)	.043*	1.33 mm	0.833	0.867
			1.90 mm	0.500	0.933
**BWT decrease T1 (%)**	0.744 (0.47-1.00)	.087	31.88%	0.667	0.800
			24.72%	0.833	0.733
**Submucosa T1 (mm)**	0.756 (0.55-0.97)	.073	1.04 mm	0.667	0.733
**Submucosa decrease T1 (mm)**	0.756 (0.51-0.99)	.073	0.59 mm	0.667	0.800
**Submucosa decrease T1 (%)**	0.800 (0.58-1.00)	.036*	35.44%	0.667	0.867
			20.76%	0.833	0.733
**UC-IUS T1**	0.850 (0.68-1.00)	.014*	< 5 points	0.833	0.667
**UC-IUS decrease T1**	0.822 (0.54-1.00)	.024*	≥3 points	0.833	0.933
**SWE T1 (kPa)**	0.589 (0.34-0.83)	.533	34.63 kPa	0.500	0.667
**SWE change T1 (kPa)**	0.678 (0.43-0.92)	.213	1.84 kPa	0.667	0.600
			8.50 kPa	0.500	0.867
**RSE T1 (grayscale value)**	0.607 (0.34-0.87)	.458	86.91	0.643	0.667
**Sigmoid T2**					
**BWT decrease T2 (mm)**	0.933 (0.81-1.00)	.002*	1.06 mm	1.000	0.933
**BWT decrease T2 (%)**	0.933 (0.81-1.00)	.002*	32.87%	1.000	0.933
**Submucosa decrease T2 (mm)**	0.933 (0.81-1.00)	.002*	0.51 mm	1.000	0.933
**Submucosa decrease T2 (%)**	0.933 (0.81-1.00)	.002*	37.27%	1.000	0.933
**UC-IUS T2**	0.811 (0.63-0.99)	.029*	<4 points	0.833	0.600
**UC-IUS decrease T2**	0.906 (0.77-1.00)	.004*	≥4 points	0.833	0.867
**SWE change T2 (kPa)**	0.633 (0.36-0.91)	.350	8.71 kPa	0.800	0.500
**RSE T2 (grayscale value)**	0.762 (0.47-1.00)	.070	74.79	0.833	0.867

Abbreviations: ROC, receiver operator characteristic; AUC, area under the curve; sens, sensitivity; spec, specificity; BWT, bowel wall thickness; UC-IUS, ulcerative colitis intestinal ultrasound index; SWE, shear-wave elastography; RSE, relative submucosal echogenicity; T0, baseline; T1, week 4; T2, follow-up endoscopy.*P < .05

### 3.3. Changes in IUS parameters at T2

#### 3.3.1. BWT and submucosal thickness at T2 in endoscopic responders

BWT was significantly lower in endoscopic responders compared to non-responders at T2 (2.09 mm [1.8-2.5] vs 4.10 mm [2.2-5.6], *P *= .023). Additionally, millimeter and percentage decrease were significantly higher in endoscopic responders at T2 (2.10 ± 0.6 mm vs 0.47 ± 1.4 mm; *P *= .006 and 48.4 ± 10.2% vs 10.7 ± 25.9%; *P *= .001, respectively; Table 2).

Although the median submucosal thickness in endoscopic responders at T2 was not significantly lower (0.73 mm [0.7-1.0] vs 1.93 [0.8-2.6], *P *= .055), the decrease in millimeter and percentage of the submucosal thickness compared to T0 was significantly different between the two groups (0.88 ± 0.3 mm vs 0.23 ± 0.5 mm; *P *= .007 and 51.3 ± 10.3% vs 12.8 ± 23.6%; *P *= .001, respectively; Table 2). The mucosa and the muscularis propria were also significantly different between endoscopic responders and non-responders at T2 (Figure 2).

#### 3.3.2. Other B-mode parameters of the sigmoid at T2 in endoscopic responders

At T2, all endoscopic responders (*n* = 6) had no hyperemia in the sigmoid colon, while in the non-responders only four patients reached an mLimberg score of 0 (100% vs 27%, *P *= .004; Table 3). Looking at change in haustration, more patients in the endoscopic response group had a normalization of their haustration compared to non-responders at T2 (67% vs 13%, *P *= .031; Table 3). All other B-mode parameters in the sigmoid were not significantly different between the two groups at this time point (Table 3).

#### 3.3.3. Optimal cut-offs at T2 associated with endoscopic response

Both a millimeter decrease of BWT of ≥1.06 mm and a percentage decrease of BWT of ≥32.87% at T2 demonstrated endoscopic response in the sigmoid with 100% sensitivity, 93% specificity, 86% PPV, and 100% NPV (AUROC: 0.93, 95% CI 0.81-1.00, *P *= .002; Table 4). A percentage decrease of ≥37.27% in submucosal thickness at T2 was accurate to determine endoscopic response with 100% sensitivity, 93% specificity, 86% PPV, and 100% NPV (AUROC: 0.93, 95% CI 0.81-1.00, *P *= .002; Table 4). Additionally, a decrease of ≥4 points in the UC-IUS index at T2 compared to T0 demonstrated endoscopic response with 83% sensitivity, 87% specificity, 71% PPV, and 93% NPV (AUROC: 0.91, 95% CI 0.77-1.00, *P *= .004; Table 4).

### 3.4. Logistic regression for IUS parameters

#### 3.4.1. IUS parameters at T1 predicting endoscopic response

In univariate analyses a BWT decrease of ≥1.33 mm (OR: 32.5 [2.38-443.15], *P *= .009; Table 5) or ≥24.72% at T1 in the sigmoid (OR 13.8 [1.21-156.6], *P *= .035; Table 5) predicted endoscopic response. At T1, a percentage decrease of the submucosal thickness in the sigmoid of ≥20.76% predicted endoscopic response (OR: 13.8, [1.21-156.6], *P *= .035; Table 5). At T1, CDS per one category decrease, and a decrease of at least one category compared to T0 were significant predictors of endoscopic response (OR 3.86 [1.29-11.49]; *P *= .015, and OR 20.0 [1.66-241.7]; *P *= .018, respectively; Table 5).

**Table 5.** Logistic regression for endoscopic response (EMS decrease of ≥1) at T1.

	Univariable	
Sigmoid T1	Odds ratio (95% CI)	*P*-value
**BWT (per mm decrease)**	2.94 (0.85-10.10)	.087
**BWT (per % decrease)**	1.03 (0.99-1.08)	.118
**BWT ≤3.22 mm**	4.00 (0.54-29.81)	.176
**BWT decrease ≥1.33 mm**	32.5 (2.38-443.15)	.009*
**BWT decrease ≥24.72%**	13.8 (1.21-156.6)	.035*
**Submucosa (per mm decrease)**	20.8 (0.81-500.0)	.067
**Submucosa (per % decrease)**	1.06 (1.00-1.12)	.049*
**Submucosa ≤1.04 mm**	5.50 (0.71-42.6)	.103
**Submucosa decrease ≥0.59 mm**	8.00 (0.96-66.5)	.054
**Submucosa decrease ≥20.76%**	13.8 (1.21-156.6)	.035*
**CDS (per one category decrease)**	3.86 (1.29-11.49)	.015*
**CDS (≥1 decrease in mLimberg)**	20.0 (1.66-241.7)	.018*
**CDS (mLimberg of 0)**	28.0 (1.99-394.4)	.014*
**Loss of stratification**	^a^	1.00
**Loss of haustration**	^a^	1.00
**Presence of fatty wrapping**	0.33 (0.05-2.43)	.279
**Presence of lymph nodes**	^a^	1.00
**UC-IUS (per point decrease)**	2.28 (1.18-4.41)	.015*
**UC-IUS <5 points**	10.0 (0.91-110.28)	.060
**UC-IUS ≥3 points decrease**	70.0 (3.65-1342.67)	.005*
**SWE (kPa)**	1.08 (0.98-1.18)	.132
**SWE (per kPa increase)**	1.03 (0.97-1.10)	.353
**SWE change ≥8.50 kPa**	6.50 (0.73-57.8)	.093
**RSE (grayscale value)**	0.99 (0.97-1.02)	.455
**RSE > 86.91 grayscale value**	0.28 (0.04-2.1)	.214

Abbreviations: T0, baseline; T1, week 4; T2, follow-up endoscopy; CI, confidence interval; BWT, bowel wall thickness; CDS, color Doppler signal; mLimberg, modified Limberg classification; IUS, intestinal ultrasound; SWE, shear-wave elastography; RSE, relative submucosal echogenicity.

a Undefined due to small sample size; in one of both groups no patient was present.*P < .05

### 3.5. Shear-wave elastography

#### 3.5.1. Responsiveness of SWE for endoscopic response

SWE in the sigmoid colon including change in SWE at T1 and T2 compared to T0 were not significantly different between endoscopic responders and non-responders based on the EMS.

**Figure 4.**
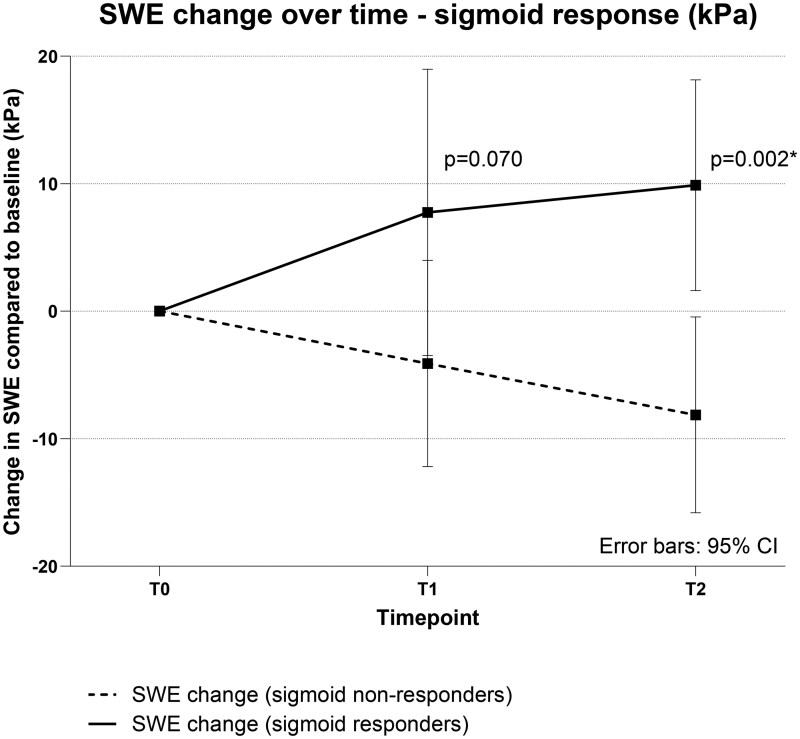
Change in SWE (kPa) over time between sigmoid responders and non-responders (endoscopic Mayo score decrease of ≥1 in the sigmoid colon). SWE, shear-wave elastography; CI, confidence interval; T0, baseline; T1, week 4; T2, follow-up endoscopy.

When response to treatment was based solely on the EMS in the sigmoid colon (ie, ≥1 decrease in EMS in the sigmoid colon), 10 patients were endoscopic responders in the sigmoid (10/21, 48%). Median SWE of the sigmoid was again not significantly different between these groups at any time points. However, the change in SWE compared to T0 showed a trend at T1 (7.7 ± 15.7 kPa vs −4.1 ± 12.0 kPa, *P *= .071) and was significantly different at T2 between sigmoid endoscopic responders and non-responders (9.9 ± 11.5 kPa vs −8.1 ± 11.4 kPa, *P *= .002; Table 6, Figure 4).

**Table 6.** SWE parameters between endoscopic responders (EMS decrease ≥1) and non-responders based on the EMS in the sigmoid colon.

Variables in the sigmoid	Sigmoid endoscopic response (*n* = 10)	Sigmoid endoscopic non-response (*n* = 11)	*P*-value
**SWE T0 in kPa; median (IQR)**	28.7 (22.9-31.5)	35.0 (29.8-47.1)	.078
**SWE T1 in kPa; median (IQR)**	33.9 (25.1-42.8)	30.6 (28.0-37.4)	.673
**SWE T2 in kPa; median (IQR)**	36.1 (31.1-46.2)	27.1 (22.3-34.8)	.057
**SWE change T1 in kPa; mean±SD**	7.7 ± 15.7	−4.1 ± 12.0	.066
**SWE change T2 in kPa; mean±SD**	9.9 ± 11.5	−8.1 ± 11.4	.002*

Abbreviations: SWE, shear-wave elastography; IQR, interquartile range; T0, baseline; T1, week 4; T2, follow-up endoscopy; SD, standard deviation.*P < .05

#### 3.5.2. Correlation of SWE with endoscopic disease activity and B-mode IUS parameters in the sigmoid

**Figure 5.**
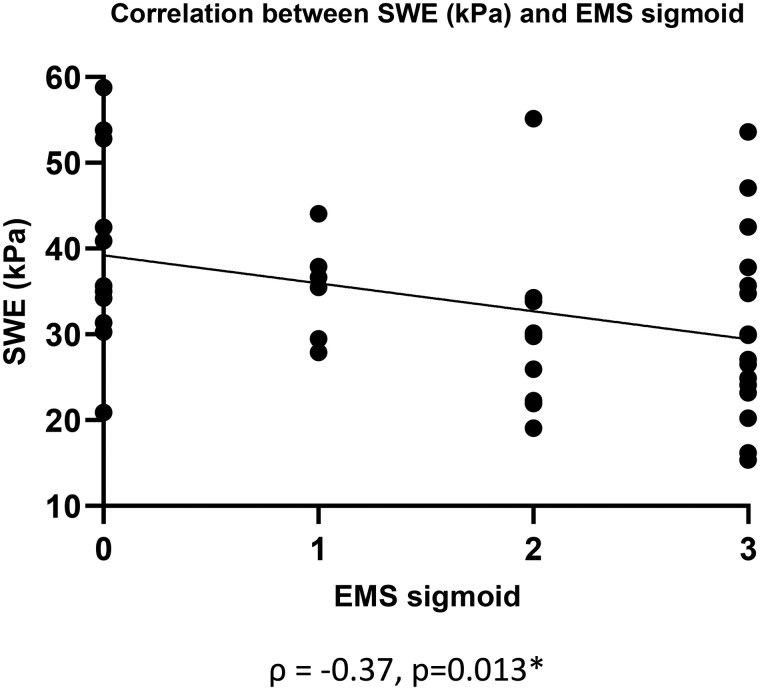
Spearman’s correlation between SWE and EMS as measured in the sigmoid colon. SWE, shear-wave elastography; EMS, endoscopic Mayo score.

There was no significant correlation between SWE and EMS for T0 and follow-up endoscopy combined (Table S7), but we observed a significant negative correlation between SWE and EMS of the sigmoid colon (Figure 5).

**Figure 6.**
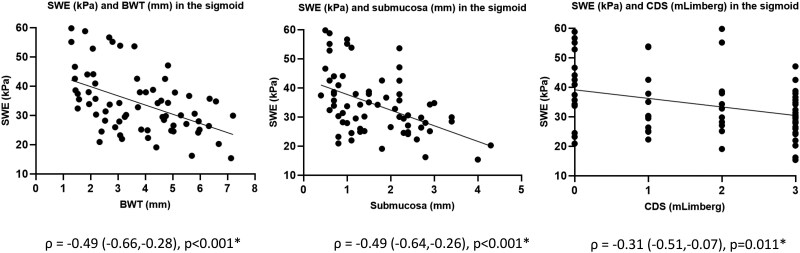
Spearman’s correlations between SWE and B-mode IUS parameters in the sigmoid colon at all time points combined. SWE, shear-wave elastography; BWT, bowel wall thickness; CDS, color Doppler signal; mLimberg, modified Limberg classification.

For IUS parameters, there were no significant correlations observed at T0. At T1, we observed a significant negative correlation between SWE and BWT, submucosal thickness, and loss of haustration (Table S8). At T2, there was a significant negative correlation between SWE and BWT, submucosal thickness, CDS, loss of haustration, and presence of fatty wrapping (Table S8). For all time points combined we observed significant negative correlations between SWE and BWT, submucosal thickness, and CDS (Figure 6).

### 3.6. Submucosal hyper-echogenicity in the sigmoid

RSE was numerically higher in endoscopic non-responders at all time points (74.9 vs 94.4, *P *= .338 at T0, 86.2 vs 101.5 at T1, *P *= .414, and 66.3 vs 98.0, *P *= .247 at T2, respectively; Table 2).

RSE at T2 above a cut-off of 74.79 grayscale value was associated with endoscopic response (OR of 0.031 [0.002-0.42], *P *= .009; Table S3). There was a significant negative correlation between RSE and the EMS (Table S7).

### 3.7. Interobserver agreement for IUS parameters

Interobserver agreement was excellent for BWT (ICC: 0.91; 95% CI, 0.78-0.96; *P *< .001) and good for submucosal thickness (ICC: 0.86; 95% CI, 0.70-0.82; *P *< .001) in the sigmoid colon (Table S9). Interobserver agreement for the other IUS parameters is presented in Table S9.

## 4. Discussion

In this prospective, observational study of moderate to severe UC patients, we demonstrated that SWE values were inversely correlated with B-mode parameters and differed significantly between responders and non-responders to filgotinib, although they did not predict treatment response at an early time point. This is the first study to assess SWE values over time in relation to treatment response in UC. Among responders in our cohort, SWE values increased over time, suggesting that the resolution of inflammation and edema results in relatively stiffer bowel walls.^17^ In contrast, SWE values in non-responders plateaued or decreased, reflecting persistent or worsening edema.

In Crohn’s disease (CD), one study did not find changes in SWE values over time, but demonstrated lower SWE values in responders at baseline.^24^ Conversely, another relatively small study involving CD patients treated with anti-TNF reported a decrease of SWE values over time in responders.^19^ In CD studies, SWE has mostly been used to assess fibrosis, showing variable results.^25^ Although chronic inflammation in UC involves submucosal changes such as fibrosis and fat deposition,^7^ we did not find any differences in SWE values based on biologic exposure, disease duration, or age. Moreover, no correlation was found between SWE and RSE, a quantitative measure of submucosal echogenicity that serves as a marker of fat deposition within the submucosa.^8^ These findings do not support a relationship between SWE values and disease chronicity in UC.

In this study, B-mode parameters measured in the sigmoid colon predicted endoscopic response assessed in the most severely affected segment. In contrast, SWE measurements in the sigmoid colon reflected only the local response within the sigmoid, making it less reliable for evaluating overall disease activity. Therefore, despite its dynamics, SWE appears less practical in clinical care compared to standard B-mode parameters. Since SWE must be performed in real-time and cannot be adjusted later, evaluating all segments is time consuming.^21^ Moreover, when the bowel wall normalizes, accurate measurements become more challenging due to the thinner bowel wall; it is also uncertain how the surrounding tissue, for example persisting fat wrapping, affects these measurements.

We demonstrate that a decrease in BWT of the sigmoid at 4 weeks of ≥1.3 mm or ≥25% compared to baseline accurately predicts endoscopic response to filgotinib treatment. In a recent study, changes in BWT at 6 weeks were found to predict endoscopic improvement in the sigmoid in UC patients treated with anti-inflammatory treatments.^13^ When assessed for individual therapeutic agents, tofacitinib demonstrated the fastest response on IUS, primarily in the submucosa, though patient numbers were limited.^13^ Our study builds on this observation by demonstrating that filgotinib treatment is also able to induce IUS changes as early as week 4, not only reflecting a rapid treatment effect, but also serving as a predictor of endoscopic response.

Moreover, a consensus study defined treatment response on IUS in UC as a reduction in BWT of >25% or >2.0 mm or >1.0 mm combined with a one-grade reduction in CDS.^26^ These numbers are also reported in other studies investigating various advanced therapies.^11^^,^^13^^,^^27^ In our study, we found that similar criteria were predictors of endoscopic response at 4 weeks after starting filgotinib treatment.

We also confirm that the submucosa is the most responsive layer to medical interventions at an early time point.^13^ Additionally, changes in CDS were also already observed at week 4. Since most vessels and, consequently, edema are present in the submucosa, early treatment effects may be most visible using these IUS parameters. Interestingly, at T2 this distinction disappeared, with both BWT and submucosal thickness showing similar trends across all endpoints.

For clinical response, CDS was the only significant predictor at T1. A decrease in BWT at T1 may be too early to distinguish clinical responders from non-responders, or BWT might be less representative for clinical treatment success compared to CDS. These findings are in line with earlier studies.^13^

No IUS parameters were associated with biochemical remission in this study. This is largely attributable to the small sample size, as well as missing data, high variability in FCP levels, and the fact that none of the patients achieved endoscopic remission. As a result, residual inflammation could have contributed to persistently elevated FCP values.

Our study was powered to assess endoscopic improvement rather than remission. Although none of the patients reached endoscopic remission, the proportions found in our cohort remain representative, as they were consistent with the phase 2/3 SELECTION trial.^15^ In this study, the biologic-experienced group (two or more previous biologics in 69% vs 61% in our cohort) demonstrated comparable rates of clinical and endoscopic remission at week 10 to those observed in our study (11.5% vs 14% for clinical remission by MMS and 3.4% vs 0% for endoscopic remission).

Our study had several limitations. First, although we performed a power calculation based on BWT changes in patients with endoscopic improvement, the sample size was too small to assess other parameters or endpoints. Additionally, the small sample size limited the ability to perform multivariate analysis; therefore, the observed predictive value of individual IUS parameters may be influenced by confounding factors. Second, due to the observational nature of the study, a relatively high proportion of patients used concomitant corticosteroids, and there was considerable variability in the timing of the follow-up endoscopy at T2. On the other hand, this reflects real-world clinical practice. Third, as transabdominal assessment of the rectum is not feasible, assessing only the sigmoid may miss patients with persisting proctitis. Despite these limitations, our study has several strengths. We used a prospective observational study design with an early time point, and endoscopic procedures were centrally read by blinded readers.

In conclusion, our study demonstrated IUS kinetics in UC patients treated with filgotinib. We observed that SWE values in the sigmoid colon change over time and differed between endoscopic responders and non-responders. Importantly, B-mode IUS was able to predict endoscopic response as early as week 4, whereas SWE values did not show predictive value at that time point. These findings highlight the value of IUS as an early surrogate marker of treatment response and suggest that although SWE provides additional insights into disease activity, its role in treatment monitoring remains limited.

## Supplementary Material

jjaf185_Supplementary_Data

## Data Availability

The data underlying this article are available in the article and in its online supplementary material.
