# Supplementary material for: Kinetics of intestinal ultrasound and shear-wave elastography to assess early response in ulcerative colitis patients treated with filgotinib
Source: J Crohns Colitis. 2025 Oct 28;19(11):jjaf185. doi: 10.1093/ecco-jcc/jjaf185 (PMC12700646; doi:10.1093/ecco-jcc/jjaf185)
Supplement: jjaf185_Supplementary_Data [file jjaf185_supplementary_data.zip › Supplementary Table 1.docx]

| **IUS B-mode parameter** | **Measurement** |
| --- | --- |
| Bowel wall thickness (BWT) | 4 measurements from lumen-mucosa interface up to muscularis propria-serosa interface (2× measurement longitudinal (≥10 mm between measurements) + 2× measurement cross-sectional plane (≥90° between measurements))/4 |
| Colour Doppler signal (CDS) | Categories  0: no Doppler signal (mLimberg 0)  1: 1 or 2 single vessels (mLimberg 1)  2: >2 single vessels or stretches limited to the wall (mLimberg 2)  3: stretches extending into the mesentery (mLimberg 3) |
| Haustrations | Categories  1: preserved haustrations  2: loss of haustrations  3: uncertain |
| Wall layer stratification | Categories  1: preserved wall layer stratification  2: uncertain  3: focal loss of wall layer stratification (≤3 cm)  4: extensive loss of wall layer stratification (>3 cm) |
| Fatty wrapping | Categories  1: absence of fatty wrapping  2: uncertain  3: presence of fatty wrapping |
| Mesenteric lymph nodes | Categories  1: presence of lymph nodes  2: absence of lymph nodes  3: uncertain |
| Relative submucosal echogenicity (RSE) | Difference in grayscale values (0-255) in longitudinal mean areal grayscale values measurements between the submucosa and muscularis propria in a perpendicular fashion (RSE = mean areal grayscale values submucosa – mean areal grayscale values muscularis propria) |

SUPPLEMENTARY TABLE 1: intestinal ultrasound B-mode parameters [IUS: intestinal ultrasound; mLimberg: modified Limberg classification]
