# Supplementary material for: Kinetics of intestinal ultrasound and shear-wave elastography to assess early response in ulcerative colitis patients treated with filgotinib
Source: J Crohns Colitis. 2025 Oct 28;19(11):jjaf185. doi: 10.1093/ecco-jcc/jjaf185 (PMC12700646; doi:10.1093/ecco-jcc/jjaf185)
Supplement: jjaf185_Supplementary_Data [file jjaf185_supplementary_data.zip › Supplementary Table 2.docx]

| **Endpoints** | **Definition** | **N (%)** |
| --- | --- | --- |
| Clinical response (MMS) | Decrease of ≥3 at T2 compared to T0 | 9/21 (43%) |
| Clinical remission (MMS) | ≤2 with all sub scores ≤1 at T2 | 3/21 (14%) |
| Biochemical remission | CRP ≤5 mg/L + FCP ≤150 mg/kg at T2 | 3/19 (16%) |
| Endoscopic response (EMS) | Decrease of ≥1 at T2 compared to T0 | 6/21 (29%) |
| Endoscopic improvement (EMS) | 0 or 1 at T2 | 4/21 (19%) |
| Endoscopic remission (EMS) | 0 at T2 | 0/21 (0%) |

SUPPLEMENTARY TABLE 2: all study endpoints [MMS: modified mayo score; FCP: fecal calprotectin; EMS: endoscopic mayo score; T0: baseline; T1: week 4; T2: follow-up endoscopy]
