# Supplementary material for: Kinetics of intestinal ultrasound and shear-wave elastography to assess early response in ulcerative colitis patients treated with filgotinib
Source: J Crohns Colitis. 2025 Oct 28;19(11):jjaf185. doi: 10.1093/ecco-jcc/jjaf185 (PMC12700646; doi:10.1093/ecco-jcc/jjaf185)
Supplement: jjaf185_Supplementary_Data [file jjaf185_supplementary_data.zip › Supplementary Table 3 (revisions).docx]

| **Logistic regression for endoscopic response T2** | Univariable |  |
| --- | --- | --- |
| **Sigmoid T2** | **Odds-ratio (95% CI)** | **P-value** |
| BWT (per mm decrease) | 3.13 (1.02-10.0) | **0.045** |
| BWT (per % decrease) | 1.09 (1.01-1.18) | **0.028** |
| BWT decrease ≥32.87% | ^a^ | 1.00 |
| Submucosa (per mm decrease) | 24.4 (1.20-500.0) | **0.038** |
| Submucosa (per % decrease) | 1.10 (1.01-1.91) | **0.025** |
| Submucosa decrease ≥37.27% | ^a^ | 1.00 |
| CDS (per one category decrease) | ^a^ | 1.00 |
| CDS (≥1 decrease in mLimberg) | ^a^ | 1.00 |
| CDS (mLimberg of 0) | ^a^ | 1.00 |
| Loss of stratification | 2.80 (0.15-53.7) | 0.495 |
| Loss of haustration | 0.15 (0.02-1.88) | 0.150 |
| Presence of fatty wrapping | 0.13 (0.01-1.44) | 0.097 |
| Presence of lymph nodes | ^a^ | 1.00 |
| UC-IUS (per point decrease) | 2.24 (1.13-4.46) | **0.021** |
| UC-IUS < 4 points | 7.5 (0.69-81.25) | 0.097 |
| UC-IUS ≥ 4 points decrease | 32.5 (2.38-443.15) | **0.009** |
| SWE (kPa) | 1.07 (0.97-1.18) | 0.194 |
| SWE (per kPa increase) | 1.03 (0.96-1.10) | 0.411 |
| RSE (grayscale value) | 0.98 (0.95-1.01) | 0.138 |
| RSE > 74.79 grayscale value | 0.031 (0.002-0.42) | **0.009** |

SUPPLEMENTARY TABLE 3: Logistic regression for endoscopic response (EMS decrease of ≥1) T2 (T0: baseline; T1: week 4; T2: follow-up endoscopy; CI: confidence interval; BWT: bowel wall thickness; CDS: Colour Doppler Signal; mLimberg: modified Limberg classification; IUS: intestinal ultrasound; SWE: shear-wave elastography; RSE: relative submucosal echogenicity)

^a^undefined due to small sample size, in one of both groups no patient was present

*IUS parameters at T2 associated with endoscopic response*

At T2, univariate analyses demonstrated that BWT in the sigmoid per mm and per % decrease was associated with endoscopic response (OR 3.13 [1.02-10.0], p=0.045 and 1.09 [1.01-1.18, p=0.028, respectively; **Supplementary** **Table 3**). Change in submucosal thickness in the sigmoid per mm and per % decrease was associated with endoscopic response (OR 24.4 [1.20-500.0], p=0.038 and 1.10 [1.01-1.91, p=0.025, respectively; **Supplementary** **Table 3**).
