# Supplementary material for: Kinetics of intestinal ultrasound and shear-wave elastography to assess early response in ulcerative colitis patients treated with filgotinib
Source: J Crohns Colitis. 2025 Oct 28;19(11):jjaf185. doi: 10.1093/ecco-jcc/jjaf185 (PMC12700646; doi:10.1093/ecco-jcc/jjaf185)
Supplement: jjaf185_Supplementary_Data [file jjaf185_supplementary_data.zip › Supplementary Table 4 (revisions).docx]

| **Logistic regression for clinical response (MMS decrease of ≥3)** | Univariable |  |
| --- | --- | --- |
| **Sigmoid T1** | **Odds-ratio (95% CI)** | **P-value** |
| BWT (per mm decrease) | 1.77 (0.70-4.46) | 0.224 |
| BWT (per % decrease) | 1.02 (0.98-1.05) | 0.353 |
| Submucosa (per mm decrease) | 1.50 (0.22-10.10) | 0.678 |
| Submucosa (per % decrease) | 1.00 (0.97-1.04) | 0.819 |
| CDS (per one category decrease) | 2.42 (1.00-5.26) | 0.051 |
| CDS (≥1 decrease in mLimberg) | 10.0 (1.3-78.1) | **0.028** |
| CDS (mLimberg of 0) | 8.8 (0.77-100.2) | 0.080 |
| Loss of stratification | ^a^ | 1.000 |
| Loss of haustration | 0.40 (0.06-2.80) | 0.356 |
| Presence of fatty wrapping | 0.25 (0.04-1.56) | 0.138 |
| Presence of lymph nodes | 1.38 (0.07-25.43) | 0.831 |
| UC-IUS (per point decrease) | 1.42 (0.91-2.23) | 0.127 |
| SWE (per kPa increase) | 1.004 (0.95-1.07) | 0.897 |
| SWE (kPa) | 1.005 (0.92-1.09) | 0.904 |
| RSE (grayscale value) | 0.98 (0.96-1.00) | 0.159 |
| **Sigmoid T2** | **Odds-ratio (95% CI)** | **P-value** |
| BWT (per mm decrease) | 4.61 (1.15-4.61) | **0.031** |
| BWT (per % decrease) | 1.06 (1.01-1.12) | **0.024** |
| Submucosa (per mm decrease) | 61.2 (1.19-3121.9) | **0.040** |
| Submucosa (per % decrease) | 1.06 (1.01-1.12) | **0.024** |
| CDS (per one category decrease) | 1.85 (0.88-3.85) | 0.106 |
| CDS (≥1 decrease in mLimberg) | 2.00 (0.33-11.97) | 0.448 |
| CDS (mLimberg of 0) | 10.50 (1.4-81.1) | **0.024** |
| Loss of stratification | a | 1.000 |
| Loss of haustration | 0.204 (0.03-1.43) | 0.109 |
| Presence of fatty wrapping | 0.357 (0.06-2.16) | 0.262 |
| Presence of lymph nodes | 0.625 (0.05-8.20) | 0.720 |
| UC-IUS (per point decrease) | 1.59 (1.02-2.50) | **0.043** |
| SWE (per kPa increase) | 1.053 (0.98-1.13) | 0.153 |
| SWE (kPa) | 1.098 (0.99-1.22) | 0.085 |
| RSE (grayscale value) | 0.986 (0.96-1.01) | 0.217 |

SUPPLEMENTARY TABLE 4: Logistic regression for clinical response (MMS decrease of ≥3) [T0: baseline; T1: week 4; T2: follow-up endoscopy; CI: confidence interval; BWT: bowel wall thickness; CDS: Colour Doppler Signal; mLimberg: modified Limberg classification; IUS: intestinal ultrasound; SWE: shear-wave elastography; RSE: relative submucosal echogenicity].

^a^undefined due to small sample size, in one of both groups no patient was present

*IUS parameters predicting clinical endpoints*

At T1, only CDS significantly predicted clinical response (CDS, ≥1 decrease in mLimberg: OR 10.0 [1.3-78.1], p=0.028; **Supplementary Table 4**). BWT, submucosal thickness, CDS and presence of fatty wrapping at T2 were significantly associated with clinical response (**Supplementary Table 4**).
