# Supplementary material for: Kinetics of intestinal ultrasound and shear-wave elastography to assess early response in ulcerative colitis patients treated with filgotinib
Source: J Crohns Colitis. 2025 Oct 28;19(11):jjaf185. doi: 10.1093/ecco-jcc/jjaf185 (PMC12700646; doi:10.1093/ecco-jcc/jjaf185)
Supplement: jjaf185_Supplementary_Data [file jjaf185_supplementary_data.zip › Supplementary_Table_5_(revisions).docx]

| **Logistic regression for clinical remission (MMS ≤2, all subscores ≤1)** | Univariable |  |
| --- | --- | --- |
| **Sigmoid T1** | **Odds-ratio (95% CI)** | **P-value** |
| BWT (per mm decrease) | 1.11 (0.34-3.57) | 0.864 |
| BWT (per % decrease) | 1.001 (0.95-1.05) | 0.965 |
| Submucosa (per mm decrease) | 1.63 (0.10-25.6) | 0.727 |
| Submucosa (per % decrease) | 1.02 (0.97-1.07) | 0.474 |
| CDS (per one category decrease) | 1.16 (0.43-3.13) | 0.769 |
| CDS (≥1 decrease in mLimberg) | 4.00 (0.30-53.47) | 0.295 |
| CDS (mLimberg of 0) | 1.75 (0.12-24.7) | 0.678 |
| Loss of stratification | ^a^ | 1.000 |
| Loss of haustration | ^a^ | 1.000 |
| Presence of fatty wrapping | ^a^ | 1.000 |
| Presence of lymph nodes | ^a^ | 1.000 |
| UC-IUS (per point decrease) | 1.19 (0.70-2.03) | 0.515 |
| SWE (per kPa increase) | 0.980 (0.89-1.07) | 0.661 |
| SWE (kPa) | 0.984 (0.86-1.12) | 0.806 |
| RSE (grayscale value) | 1.002 (0.97-1.03) | 0.888 |
| **Sigmoid T2** | **Odds-ratio (95% CI)** | **P-value** |
| BWT (per mm decrease) | 1.55 (0.66-3.69) | 0.314 |
| BWT (per % decrease) | 1.04 (0.98-1.10) | 0.189 |
| Submucosa (per mm decrease) | 2.26 (0.24-21.74) | 0.479 |
| Submucosa (per % decrease) | 1.02 (0.97-1.08) | 0.341 |
| CDS (per one category decrease) | 1.22 (0.33-2.21) | 0.741 |
| CDS (≥1 decrease in mLimberg) | 1.60 (0.45-8.33) | 0.720 |
| CDS (mLimberg of 0) | 2.50 (0.19-32.8) | 0.485 |
| Loss of stratification | ^a^ | 1.000 |
| Loss of haustration | ^a^ | 1.000 |
| Presence of fatty wrapping | 0.500 (0.04-6.55) | 0.597 |
| Presence of lymph nodes | ^a^ | 1.000 |
| UC-IUS (per point decrease) | 1.33 (0.79-2.24) | 0.280 |
| SWE (per kPa increase) | 1.008 (0.92-1.10) | 0.854 |
| SWE (kPa) | 1.034 (0.93-1.16) | 0.551 |
| RSE (grayscale value) | 1.02 (0.99-1.05) | 0.273 |

SUPPLEMENTARY TABLE 5: Logistic regression for clinical remission (MMS ≤2, all subscores ≤1) [T0: baseline; T1: week 4; T2: follow-up endoscopy; CI: confidence interval; BWT: bowel wall thickness; CDS: Colour Doppler Signal; mLimberg: modified Limberg classification; IUS: intestinal ultrasound; SWE: shear-wave elastography; RSE: relative submucosal echogenicity; MMS: modified mayo score].

^a^undefined due to small sample size, in one of both groups no patient was present

No IUS parameters were associated with clinical remission at both T1 and T2 (**Supplementary Table 5**).
