# Supplementary material for: Kinetics of intestinal ultrasound and shear-wave elastography to assess early response in ulcerative colitis patients treated with filgotinib
Source: J Crohns Colitis. 2025 Oct 28;19(11):jjaf185. doi: 10.1093/ecco-jcc/jjaf185 (PMC12700646; doi:10.1093/ecco-jcc/jjaf185)
Supplement: jjaf185_Supplementary_Data [file jjaf185_supplementary_data.zip › Supplementary_Table_6_(revisions).docx]

| **Logistic regression for biochemical remission (CRP ≤ 5 mg/L AND ≤ 150 mg/kg)** | Univariable |  |
| --- | --- | --- |
| **Sigmoid T1** | **Odds-ratio (95% CI)** | **P-value** |
| BWT (per mm decrease) | 726.2 (0.031-17E6) | 0.200 |
| BWT (per % decrease) | 1.14 (0.99-1.31) | 0.075 |
| Submucosa (per mm decrease) | 2074.8 (0.46-9.3E6) | 0.075 |
| Submucosa (per % decrease) | 1.33 (0.75-2.37) | 0.331 |
| CDS (per one category decrease) | 2.22 (0.66-7.69) | 0.198 |
| CDS (≥1 decrease in mLimberg) | ^a^ | 1.000 |
| CDS (mLimberg of 0) | 6.0 (0.42-85.25) | 0.186 |
| Loss of stratification | ^a^ | 1.000 |
| Loss of haustration | ^a^ | 1.000 |
| Presence of fatty wrapping | ^a^ | 1.000 |
| Presence of lymph nodes | ^a^ | 1.000 |
| UC-IUS (per point decrease) | 4.15 (0.72-23.93) | 0.111 |
| SWE (per kPa increase) | 1.09 (0.98-1.21) | 0.103 |
| SWE (kPa) | 1.18 (1.01-1.37) | **0.033** |
| RSE (grayscale value) | 0.993 (0.96-1.02) | 0.623 |
| **Sigmoid T2** | **Odds-ratio (95% CI)** | **P-value** |
| BWT (per mm decrease) | 1.53 (0.65-3.57) | 0.333 |
| BWT (per % decrease) | 1.03 (0.97-1.09) | 0.277 |
| Submucosa (per mm decrease) | 2.54 (0.26-25.0) | 0.420 |
| Submucosa (per % decrease) | 1.02 (0.97-1.08) | 0.355 |
| CDS (per one category decrease) | 2.38 (0.53-11.11) | 0.254 |
| CDS (≥1 decrease in mLimberg) | ^a^ | 1.000 |
| CDS (mLimberg of 0) | 2.571 (0.19-34.47) | 0.476 |
| Loss of stratification | 7.50 (0.33-173.3) | 0.209 |
| Loss of haustration | 0.833 (0.06-11.28) | 0.891 |
| Presence of fatty wrapping | 0.500 (0.04-6.68) | 0.600 |
| Presence of lymph nodes | ^a^ | 1.000 |
| UC-IUS (per point decrease) | 1.41 (0.81-2.44) | 0.226 |
| SWE (per kPa increase) | 1.006 (0.93-1.1) | 0.883 |
| SWE (kPa) | 1.02 (0.91-1.14) | 0.772 |
| RSE (grayscale value) | 0.982 (0.95-1.02) | 0.283 |

SUPPLEMENTARY TABLE 6: Logistic regression for biochemical remission (CRP ≤ 5 mg/L AND ≤ 150 mg/kg) [T0: baseline; T1: week 4; T2: follow-up endoscopy; CI: confidence interval; BWT: bowel wall thickness; CDS: Colour Doppler Signal; mLimberg: modified Limberg classification; IUS: intestinal ultrasound; SWE: shear-wave elastography; RSE: relative submucosal echogenicity].

^a^undefined due to small sample size, in one of both groups no patient was present

*IUS parameters predicting biochemical remission*

At T1 and T2 no B-mode IUS parameter were significantly associated with biochemical remission (**Supplementary Table 6**).
