# Supplementary material for: Kinetics of intestinal ultrasound and shear-wave elastography to assess early response in ulcerative colitis patients treated with filgotinib
Source: J Crohns Colitis. 2025 Oct 28;19(11):jjaf185. doi: 10.1093/ecco-jcc/jjaf185 (PMC12700646; doi:10.1093/ecco-jcc/jjaf185)
Supplement: jjaf185_Supplementary_Data [file jjaf185_supplementary_data.zip › Supplementary_Table_7_(revisions).docx]

| **Spearman’s Correlations for endoscopic severity (EMS)** | Total  ρ (95% CI) | p-value |
| --- | --- | --- |
| BWT sigmoid (mm) | 0.66 (0.45-0.80) | **<0.001** |
| BWT descending colon (mm) | 0.48 (0.20-0.68) | **0.001** |
| BWT transverse colon (mm) | -0.06 (-0.31-0.30) | 0.97 |
| BWT ascending colon (mm) | -0.31 (-0.56-0.01) | **0.039** |
| Submucosa sigmoid (mm) | 0.51 (0.24-0.71) | **<0.001** |
| Submucosa descending colon (mm) | 0.40 (0.11-0.63) | **0.007** |
| Submucosa transverse colon (mm) | -0.06 (-0.36-0.25) | 0.70 |
| Submucosa ascending colon (mm) | -0.27 (-0.53-0.04) | 0.074 |
| SWE sigmoid colon (kPa) | -0.11 (-0.41-0.20) | 0.46 |
| RSE sigmoid colon (grayscale value) | -0.38 (-0.61, -0.09) | **0.011** |
| CDS (mLimberg 0-3) | 0.28 (-0.02-0.54) | 0.062 |
| Loss of stratification sigmoid | 0.25 (-0.06-0.52) | 0.098 |
| Loss of haustration sigmoid | 0.54 (0.28-0.73) | **<0.001** |
| Presence of fatty wrapping sigmoid | 0.32 (0.02-0.57) | **0.033** |
| Presence of lymph nodes sigmoid | 0.18 (-0.14-0.46) | 0.249 |

SUPPLEMENTARY TABLE 7: Spearman’s correlations for endoscopic severity based on the EMS of the worst segment and IUS B-mode variables in the sigmoid for all timepoints combined. [EMS: Endoscopic mayo score; SWE: Shear-wave elastography; IUS: intestinal ultrasound; BWT: bowel wall thickness; RSE: relative submucosal echogenicity; CDS: Colour Doppler Signal].

Correlations of IUS parameters with endoscopic disease activity

The BWT and submucosal thickness of the sigmoid and descending colon correlated significant with the EMS for all timepoints combined. Additionally, loss of haustration and presence of fatty wrapping in the sigmoid were also significantly correlated with EMS (**Supplementary Table 7**).
