# Supplementary material for: Kinetics of intestinal ultrasound and shear-wave elastography to assess early response in ulcerative colitis patients treated with filgotinib
Source: J Crohns Colitis. 2025 Oct 28;19(11):jjaf185. doi: 10.1093/ecco-jcc/jjaf185 (PMC12700646; doi:10.1093/ecco-jcc/jjaf185)
Supplement: jjaf185_Supplementary_Data [file jjaf185_supplementary_data.zip › Supplementary_Table_8_(revisions).docx]

| **Spearman’s Correlations for Shear-wave elastography (SWE) in the sigmoid colon** | | | | | | |
| --- | --- | --- | --- | --- | --- | --- |
| **IUS Variables of the sigmoid colon** | T0  ρ (95% CI) | p-value | T1  ρ (95% CI) | p-value | T2  ρ (95% CI) | p-value |
| BWT (mm) | -0.07 (-0.48-0.37) | 0.764 | -0.66 (-0.85, -0.33) | **<0.001** | -0.66 (-0.85, -0.32) | **<0.001** |
| Submucosal thickness (mm) | 0.05 (-0.38-0.46) | 0.821 | -0.67 (-0.85, -0.35) | **<0.001** | -0.67 (-0.86, -0.34) | **<0.001** |
| RSE (grayscale values) | -0.14 (-0.53-0.30) | 0.523 | 0.18 (-0.28-0.57) | 0.434 | -0.15 (-0.55-0.30) | 0.500 |
| CDS (mLimberg 0-3) | -0.21 (-0.58-0.23) | 0.337 | -0.27 (-0.62-0.17) | 0.209 | -0.45 (-0.74, -0.02) | **0.038** |
| Loss of stratification | 0.31 (-0.13-0.65) | 0.148 | -0.19 (-0.57-0.25) | 0.378 | a | 1.000 |
| Loss of haustration | -0.21 (-0.58-0.24) | 0.345 | -0.53 (-0.78, -0.13) | **0.010** | -0.59 (-0.82, -0.21) | **0.004** |
| Presence of fatty wrapping | -0.29 (-0.63-0.16) | 0.186 | -0.37 (-0.68-0.07) | 0.085 | -0.46 (-0.75, -0.04) | **0.031** |
| Presence of lymph nodes | -0.09 (-0.49-0.35) | 0.684 | -0.209 (-0.58-0.23) | 0.338 | -0.41 (-0.71-0.03) | 0.060 |

SUPPLEMENTARY TABLE 8: Spearman’s correlations for SWE and IUS B-mode variables in the sigmoid per timepoint. [SWE: Shear-wave elastography; IUS: intestinal ultrasound; BWT: bowel wall thickness; RSE: relative submucosal echogenicity; CDS: Colour Doppler Signal; T0: baseline; T1: week 4; T2: follow-up endoscopy].
