# Supplementary material for: Kinetics of intestinal ultrasound and shear-wave elastography to assess early response in ulcerative colitis patients treated with filgotinib
Source: J Crohns Colitis. 2025 Oct 28;19(11):jjaf185. doi: 10.1093/ecco-jcc/jjaf185 (PMC12700646; doi:10.1093/ecco-jcc/jjaf185)
Supplement: jjaf185_Supplementary_Data [file jjaf185_supplementary_data.zip › Supplementary_Table_9_(revisions).docx]

| Inter-rater agreement | Agreement  (95% CI) | p-value |
| --- | --- | --- |
| BWT | ICC = 0.91 (0.78-0.96) | **<0.001** |
| Submucosal thickness | ICC = 0.86 (0.70-0.92) | **<0.001** |
| CDS | κ = 0.70 (0.47-0.93) | **<0.001** |
| Loss of WLS | κ = 0.51 (0.06-0.96) | **0.005** |
| Loss of haustrations | κ = 0.62 (0.34-0.90) | **<0.001** |
| Presence of LN | κ = 0.62 (0.16-1.09) | **0.001** |
| Presence of fatty wrapping | κ = 0.28 (0.05-0.51) | **0.036** |
| Relative submucosal echogenicity | ICC = 0.61 (0.25-0.82) | **<0.001** |

SUPPLEMENTARY TABLE 9: Inter-observer agreement per IUS parameter in the sigmoid [BWT: Bowel wall thickness; CDS: Colour Doppler Signal; WLS: wall layer stratification; LN: lymph nodes]
